# Supplementary material for: Single-cell total-RNA profiling unveils regulatory hubs of transcription factors
Source: Nat Commun. 2024 Jul 15;15:5941. doi: 10.1038/s41467-024-50291-3 (PMC11251146; doi:10.1038/s41467-024-50291-3)
Supplement: Supplementary file 1 — Supplementary Information [file 41467_2024_50291_MOESM1_ESM.pdf]

Supplementary Materials

Of

Single-cell Total-RNA Profiling Unveils Regulatory Hubs of Transcription Factors

Yichi Niu[1,2,*], Jiayi Luo[1,3,*], Chenghang Zong[1,2,3,4,5,6,#]

Affiliations:

[1]Department of Molecular and Human Genetics,

[2]Genetics & Genomics Program,

[3]Cancer and Cell Biology Program,

[4]Integrative Molecular and Biomedical Sciences Program

[5]Dan L Duncan Comprehensive Cancer Center,

[6]McNair Medical Institute,

Baylor College of Medicine,

One Baylor Plaza, Houston, Texas, 77030

[*]These authors contributed equally to this work

[#]Corresponding Author: Chenghang Zong, (chenghang.zong@bcm.edu)

Supplementary note 1: The potential functions of BPTF, RREB1 and TBP1 in cell cycle regulation

In the TF association network presented in **Fig. 3e**, three TFs are absent as their regulatory relationships with the other TFs were not identified through the LASSO-based analysis. This observation suggests that these three genes may regulate gene expression along cell cycle via different mechanisms, independent of the TF network studied here. For example, BPTF, a core subunit of NURF chromatin complex, has been reported to function as a co-factor of MYC to regulate gene expression[1]. Therefore, the regulatory effects of BPTF on its downstream genes may depend on the activities of other TFs, e.g., MYC. RREB1, downstream of RAS signaling pathway, binds to RAS responsive elements upon the activation of RAS signaling[2], suggesting that its activity could depend on RAS signaling during the cell cycle. TBP1, encoding the TATA-binding protein, is an important subunit of the basal transcription factor complex. Previous studies have shown that TBP1 closely associates with condensed chromosomes during mitosis, and facilitates the transcriptional reactivation after mitosis[3-5]. Therefore, TBP1 could play an important role to coordinate the general transcriptional activity and the chromosome condensation and segregation during mitosis.

Supplementary note 2: Verify the cell-cycle dependent gene expression in VASA-seq data

To verify the noncanonical CCGs identified in our data, we examined the expression patterns along the cell cycle of these genes in VASA-plate data. Firstly, we conducted the analysis at the exon level. The cells with > 20000 exon UMIs in the VASA-plate dataset were selected and ordered along the cell cycle using reCAT. To account for the variations in single-cell data, we evenly divided these cells into 6 intervals along the cell cycle, and the average expression levels were calculated for each interval. The fold changes between the highest expression level and the lowest expression level among these intervals were then calculated. All the detected genes were

then separated into three groups. The genes with FDR < 0.1 at the exon level identified in our data (i.e., Type I & Type II CCGs) were classified into canonical and noncanonical CCGs, and the rest of genes were considered as non-CCGs. As a result, both canonical and noncanonical CCGs exhibit significantly higher fold-changes than non-CCGs across different cell cycle stages (**Supplementary Fig. 14a, left panel**). To further rule out the possibility that the observed differences stemmed from the varying levels of technical noise among different genes, we examined whether the overall expression patterns were consistent between VASA-plate data and our data for the identified CCGs. To do so, we divided the single cells in our dataset into six intervals along the cell cycle using the same strategy. Next, the average expression levels within each of these intervals were calculated to represent the overall expression patterns of each gene along the cell cycle. Next, spearman correlation coefficients between VASA-plate data and our data were calculated based on the average expression levels within these intervals. As a result, an overall strong positive correlation between two datasets was observed for both canonical (average=0.707) and noncanonical (average=0.496) CCGs (**Supplementary Fig. 14a, right panel**). In contrast, only a week correlation was observed for non-CCGs (average=0.196).

The same analyses were also applied at the intron level. The genes with FDR < 0.1 at the intron level (i.e., Type I & Type III) in our data were divided into canonical and noncanonical CCGs. The rest of genes were classified as non-CCGs. The fold-changes at the intron level of each gene across different cell cycle stages were then examined. As a result, both canonical and noncanonical CCGs exhibit significantly higher fold-changes across different cell cycle stages compared to non-CCGs (**Supplementary Fig. 14b, left panel**). In addition, the expression patterns derived from VASA-seq data and our data were also highly correlated with each other for both canonical (average= 0.725) and noncanonical (average=0.521) CCGs (**Supplementary Fig. 14b, right panel**). In contrast, only a week correlation was observed for non-CCGs

(average=0.224). In summary, these observations suggest that the CCGs identified at either the exon or intron level in our data exhibit consistent cell-cycle dependent expression patterns at the respective level in VASA-seq data.

Supplementary note 3: Identification of CCGs with different FDR cutoff

In the main text, we employed FDR < 0.1 as the cutoff to identify differentially expressed genes along cell cycle. The reason to select a relaxed FDR cutoff is to facilitate the detection of the genes with moderate changes during cell cycle. As the selection of a relaxed FDR cutoff might increase the likelihood of encountering false positives, here we reanalyzed the CCGs with FDR < 0.05. Overall, 77.1% (1021 of 1325) of exon-based CCGs and 77.5% (746 of 962) of intron-based CCGs passed the criteria. We also examined the effects of applying a more stringent FDR cutoff on each type of CCGs. As a result, for Type I CCGs, 85.1% of them (332 of 390) passed exon FDR < 0.05 and 86.7% of them (338 of 390) pass intron FDR < 0.05; 73.7 % (689 of 935) of Type II CCGs and 71.3% (408 of 572) of Type III CCGs passed the more stringent FDR at exon or intron level respectively (**Supplementary Fig. 21**). In summary, the majority of CCGs identified using a relaxed FDR cutoff can satisfy a more stringent FDR threshold, suggesting the usage of a relaxed FDR in the original analysis did not introduce a significant number of false positives.

Supplementary note 4: Exploit the parameter choices for LASSO regression model

The effectiveness of using LASSO regression to capture the TF-gene links could depend on the selection of input TFs for analysis. For example, the inclusion of numerous unrelated TFs could introduce substantial noise, potentially resulting in model failure. On the other hand, employing a too stringent criterion could overlook the complex covariation between genes. Here, we systematically evaluated the effects of the selection of input TFs on the LASSO regression results by testing different criteria on cell cycle data. In total, we examined two strategies and six criteria.

The first strategy involved applying a single cutoff (e.g., $r > 0.1$) to filter out the TFs with low correlation coefficients, while the second one entailed ordering the TF based on correlation coefficients and selecting the top percentile (e.g., top 5%). Given that Type I CCGs are mainly driven by transcriptional regulation, we utilized the TF-gene links associated with Type I CCGs for the evaluation.

Firstly, we assessed the number of Type I CCGs whose expression patterns can be successfully modeled by LASSO regression using the expression patterns of input TFs with different selection criteria. As a result, we observed that the LASSO model failed for a substantial proportion of genes when a relaxed criterion (i.e., $r > 0.1$ or top 10%) was chosen (**Supplementary Fig. 22a**), which is potentially caused by the introduction of excessive noise with the inclusion of unrelated TFs. Consistently, a notably lower number of TF-gene links were captured when employing the relaxed criteria compared to other criteria (**Supplementary Fig. 22b**). On another hand, employing a stringent criterion (i.e., $r > 0.25$) did not affect the successful model fitting. However, the utilization of the stringent criterion excluded the TFs with complex covariation with the target gene from the analysis. Therefore, a notable number of TF-gene links were missed when employing a stringent criterion (**Supplementary Fig. 22b**). In contrast, the rest of criteria performed similarly and showed a higher discovery rate (**Supplementary Fig. 22a-b**). In addition, most of the TF-gene links can be consistently identified under different criteria (**Supplementary Fig. 22b**), suggesting that these TF-gene links were not false positives specific to one single parameter. In summary, we concluded that the majority of TF-gene links can be robustly captured within the appropriate parameter interval.

a

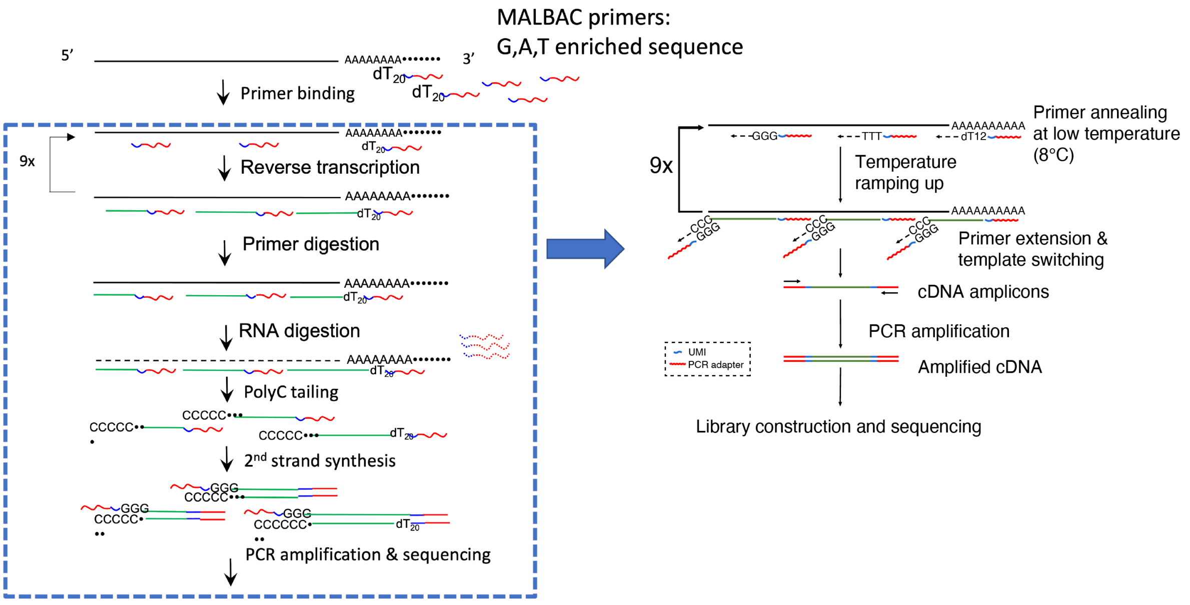

b

Method	RNA fragmentation	poly(A) tailing	Reverse transcription	Second-strand synthesis	Amplification	Strandness
snapTotal-seq	/	/	Yes (with TS)	/	PCR	/
VASA-seq	Yes	Yes	Yes	Yes	IVT	Yes
Smart-seq-total	/	Yes	Yes (with TS)	/	PCR	Yes
Smart-seq3	/	/	Yes (with TS)	/	PCR	Yes
CEL-Seq2	/	/	Yes	Yes	IVT	Yes

Supplementary figure 1. Chemistry of snapTotal-seq. (**a**) The comparison between the chemistries of MATQ-seq (left) and snapTotal-seq (right). (**b**) Summary of the major steps in snapTotal-seq, VASA-seq, Smart-seq-total, Smart-seq3 and CEL-Seq2. TS, template switching. IVT, in vitro transcription.

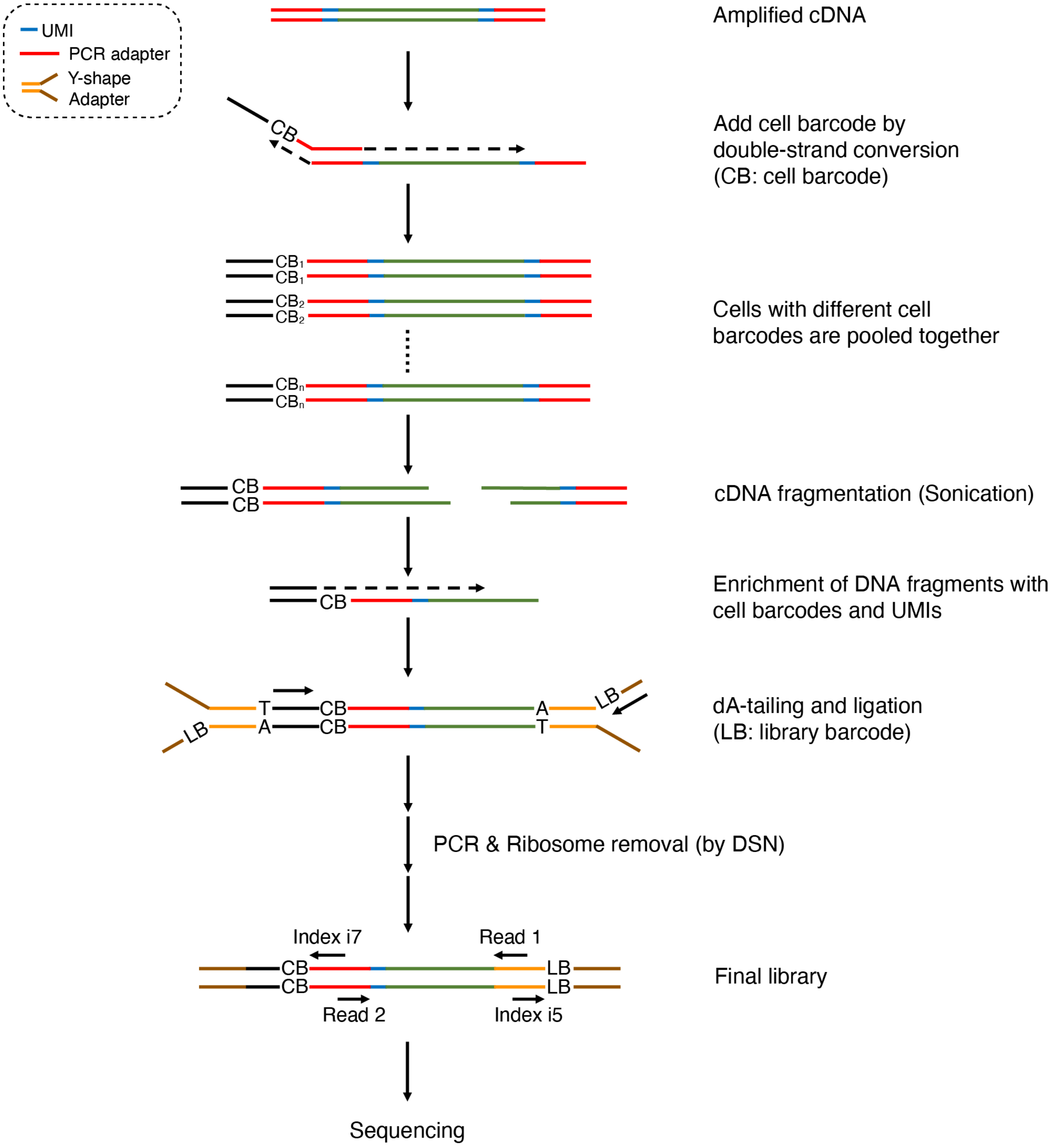

Supplementary figure 2. Scheme of library construction. The primer sequences used in snapTotal-seq are provided in **Supplementary Data 12**.

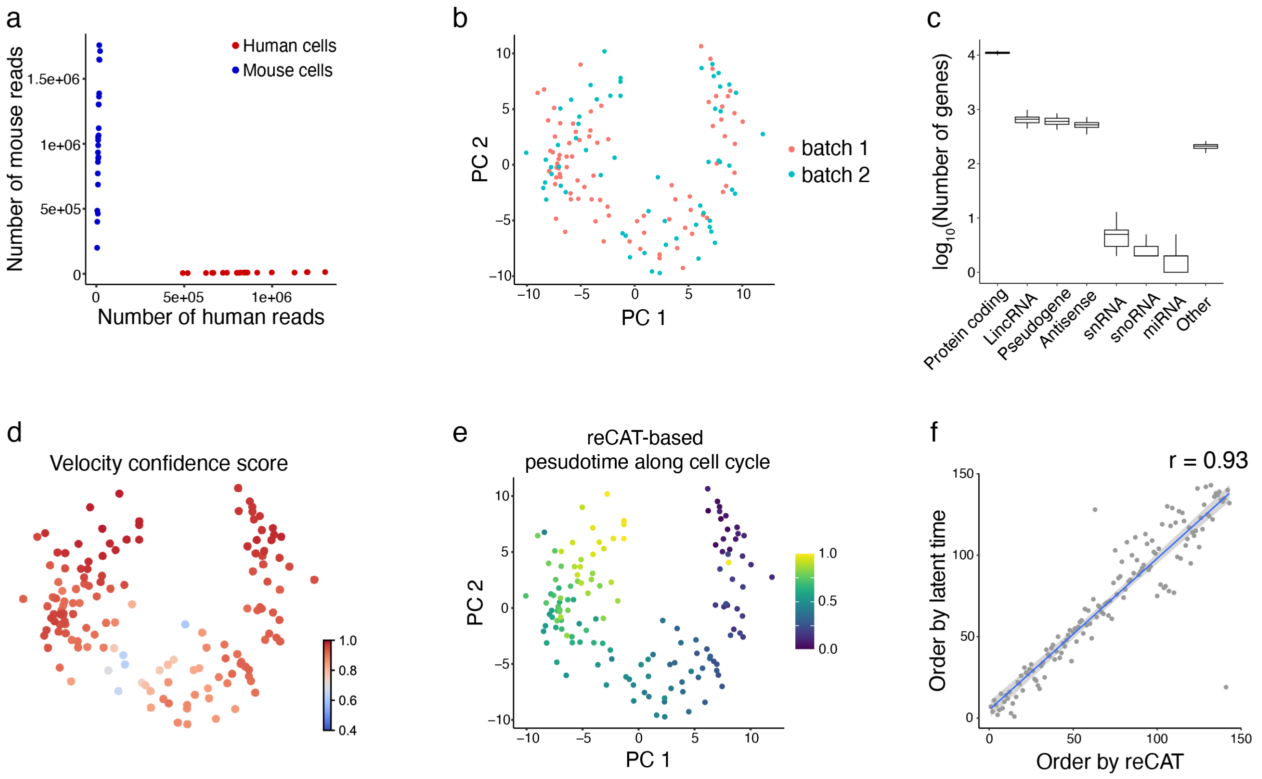

Supplementary figure 3. Benchmark analysis. (**a**) Scatter plot of the number of reads from each species detected in each single cell. (**b**) PCA plot of HEK293T cells. The cells are colored by the corresponding technical batches. (**c**) The number of genes of different biotypes detected in single cells. Center lines show median, box limits show the upper and lower quartiles, and whiskers show the 1.5× interquartile range (IQR). (**d**) Velocity confidence scores of HEK293T cells sequenced by snapTotal-seq. (**e**) The pseudo-temporal trajectory along cell cycle established by reCAT algorithm. (**f**) The scatter plot between the reCAT based cell cycle trajectory and the RNA velocity based cell cycle trajectory.

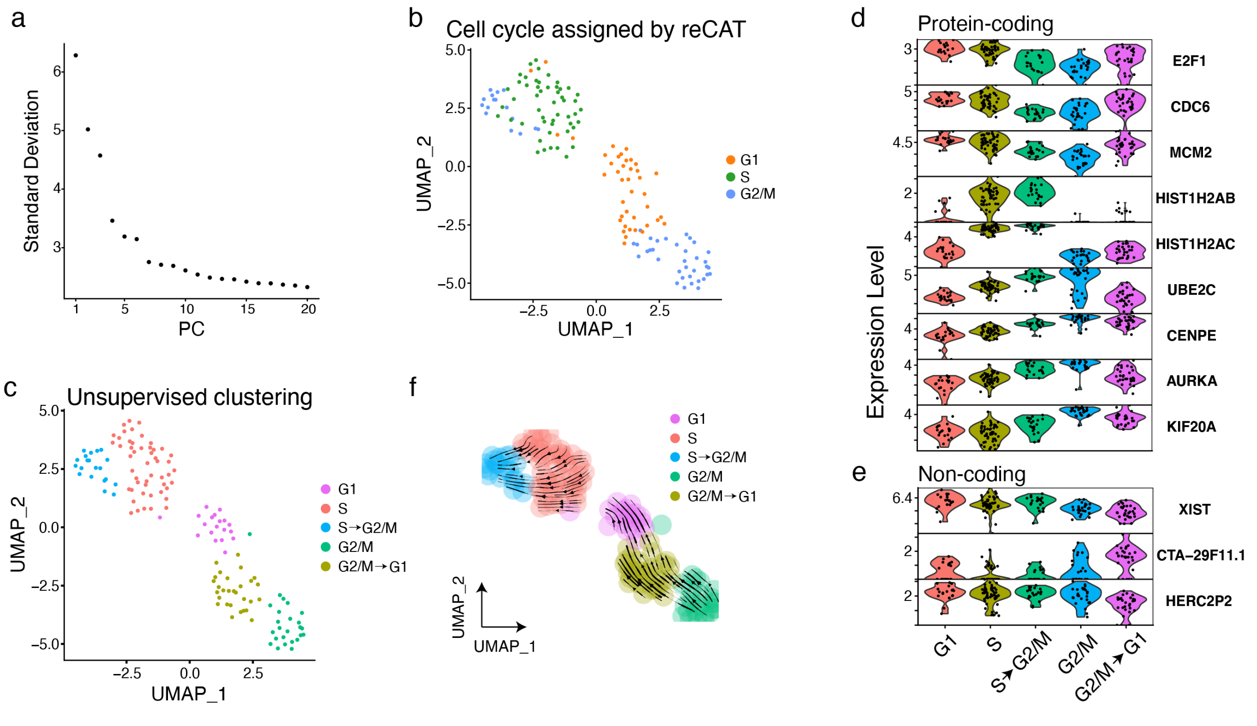

Supplementary figure 4. UMAP visualization and unsupervised clustering on snapTotal-seq data. (**a**) The ranking of principle components (PCs) based on standard deviations. The majority of true biological signal is captured in the first 6 PCs. (**b**) UMAP visualization using the first 6 PCs. (**c**) Five clusters identified by the unsupervised clustering analysis based on the first 6 PCs. (**d**) The expression of known cell-cycle marker genes across five cell clusters identified by unsupervised clustering. (**e**) The differential expression of non-coding RNAs across five cell-cycle sub-stages. (**f**) Projection of RNA velocity on UMAP plot.

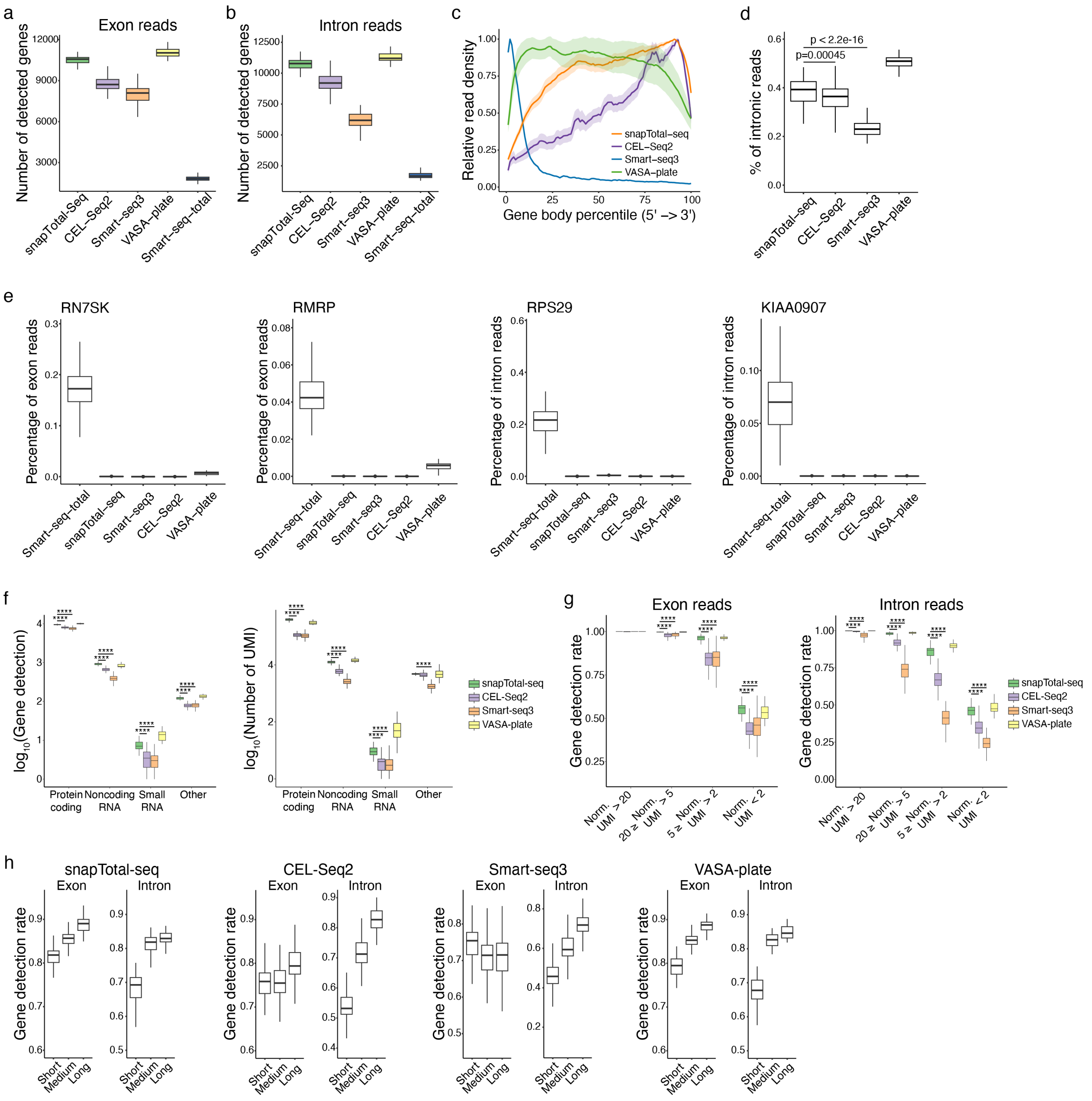

Supplementary figure 5. Gene detection sensitivity of each method. (**a-b**) The number of genes detected by exon reads or intron reads in single HEK293T cells by different methods. Panel **a**, exon reads. Panel **b**, intron reads. (**c**) The read distribution over gene body of each method. The center line and shade show the Mean ± SD. (**d**) The percentage of intron reads in each method. *P* values calculated using two-sided Student's t-test. (**e**) The dominance of the reads mapped to *RN7SK*, *RMRP*, *RPS29* and *KIAA0907* in Smart-seq-total data. (**f**) The number of genes (left) and UMI count (right) of different biotypes detected in single cells by each method. *P* values calculated using two-sided Student's t-test, **** $p < 0.0001$. (**g**) The detection rate of genes with

different expression levels by each method. *P* values calculated using two-sided Student's t-test, **** $p < 0.0001$. (**h**) The detection rate of genes with different length by each method. For equal-footing comparison, all tested cells were downsampled to the depth of 1M uniquely mapped reads per cell. For all boxplots, center lines show median, box limits show the upper and lower quartiles, and whiskers show the 1.5× interquartile range (IQR).

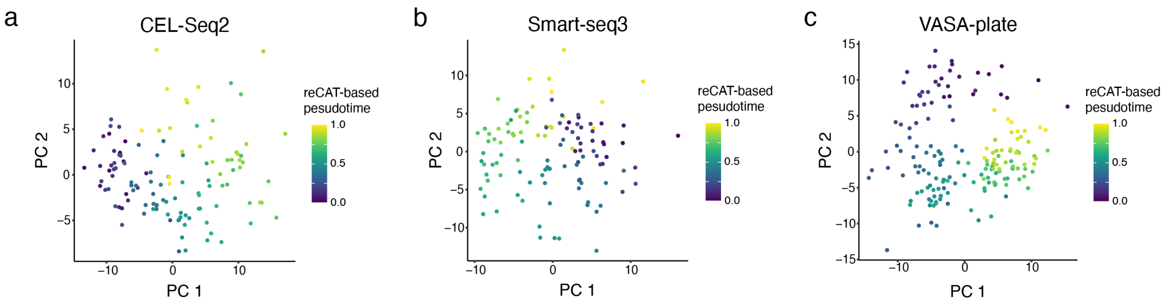

Supplementary figure 6. The cell cycle pseudotime inferred by using reCAT for CEL-Seq2 (**a**), Smart-seq3 (**b**) and VASA-plate (**c**). reCAT data for snapTotal-seq is given in **Supplementary Fig. 3e** above.

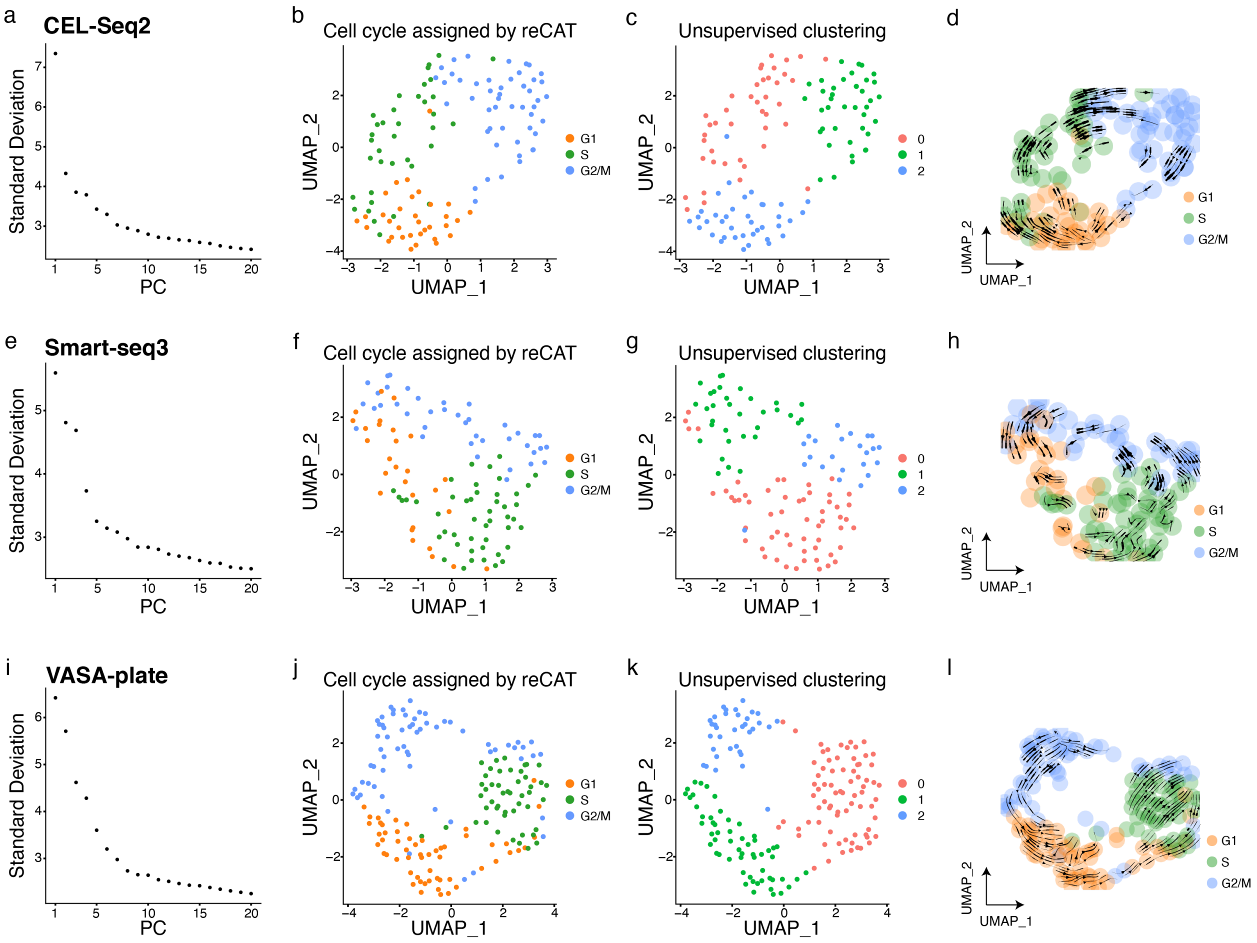

Supplementary figure 7. UMAP visualization and unsupervised clustering on CEL-Seq2, Smart-seq3 and VASA-plate data. (**a**) The ranking of principle components (PCs) based on standard deviations in CEL-Seq2 data. The majority of true biological signal is captured in the first 6 PCs. (**b**) UMAP visualization on CEL-Seq2 data using the first 6 PCs. (**c**) Unsupervised clustering based on the first 6 PCs. (**d**) Projection of RNA velocity on UMAP plot for CEL-Seq2 data. (**e**) The ranking of principle components (PCs) based on standard deviations in Smart-seq3 data. (**f**) UMAP visualization on Smart-seq3 data. The majority of true biological signal is captured in the first 8 PCs. (**g**) Unsupervised clustering based on the first 8 PCs. (**h**) Projection of RNA velocity on UMAP plot for Smart-seq3 data. (**i**) The ranking of principle components (PCs) based on standard deviations in VASA-plate data. (**j**) UMAP visualization on VASA-plate data using the first 7 PCs. (**k**) Unsupervised clustering based on the first 7 PCs. (**l**) Projection of RNA velocity on UMAP plot for VASA-plate data.

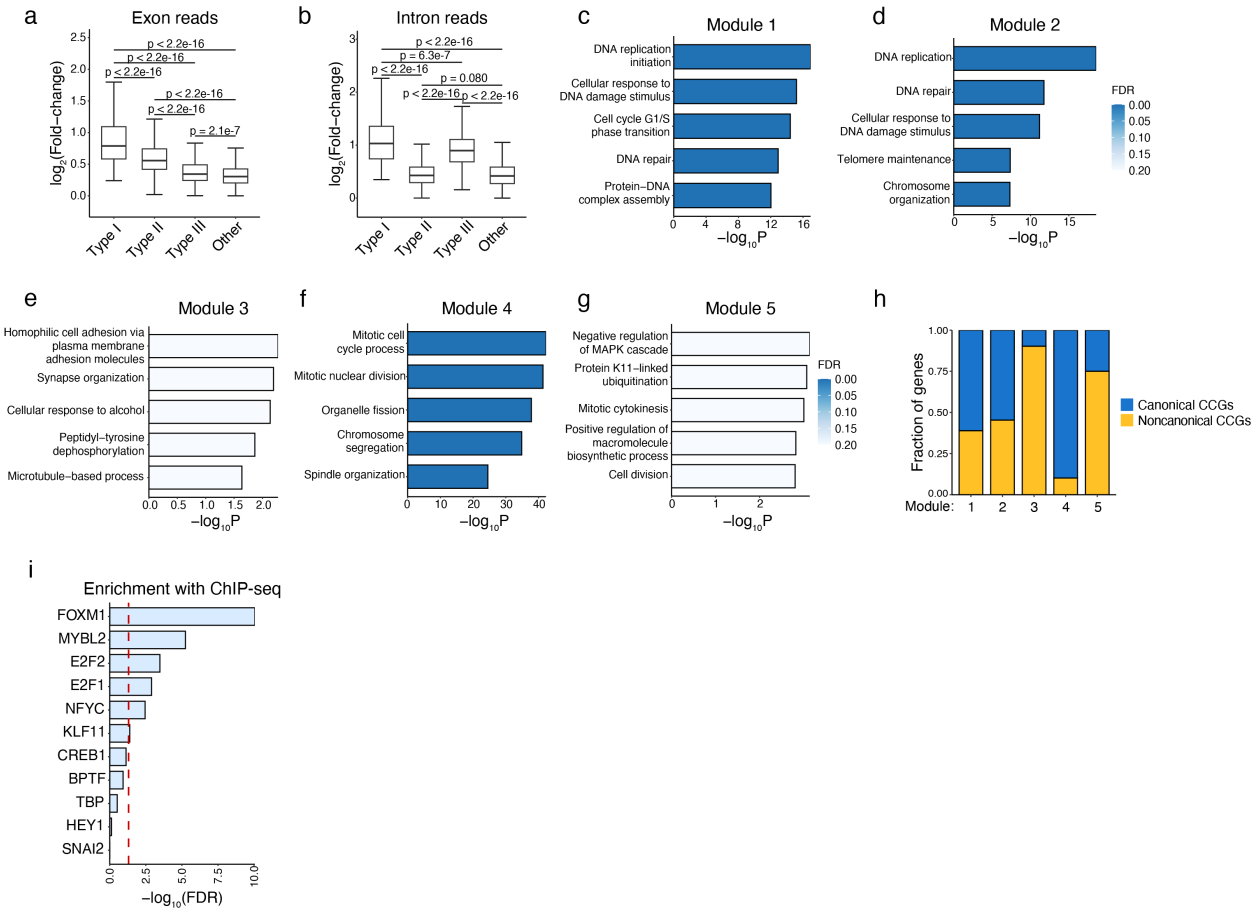

Supplementary figure 8. Type I CCGs in HEK293T cells. (**a-b**) Overall fold changes in spliced RNA (**a**) and in unspliced RNA (**b**) for different types of CCGs along cell cycle. Center lines show median, box limits show the upper and lower quartiles, and whiskers show the 1.5× interquartile range (IQR). *P* values were calculated using two-sided Wilcoxon Rank Sum test. (**c-g**) Gene Ontology (GO) enrichment analysis on Type I kinetic modules. (**h**) Fractions of noncanonical CCGs in 5 kinetic modules. Source data are provided as a Source Data file. (**i**) Enrichment analysis between the identified targets genes and the binding targets identified by ChIP-seq for each TF. Statistical significance was determined using one-sided Fisher's exact test and multiple testing correction was performed using FDR. The red dashed line corresponds to -$\log_{10}$(0.05).

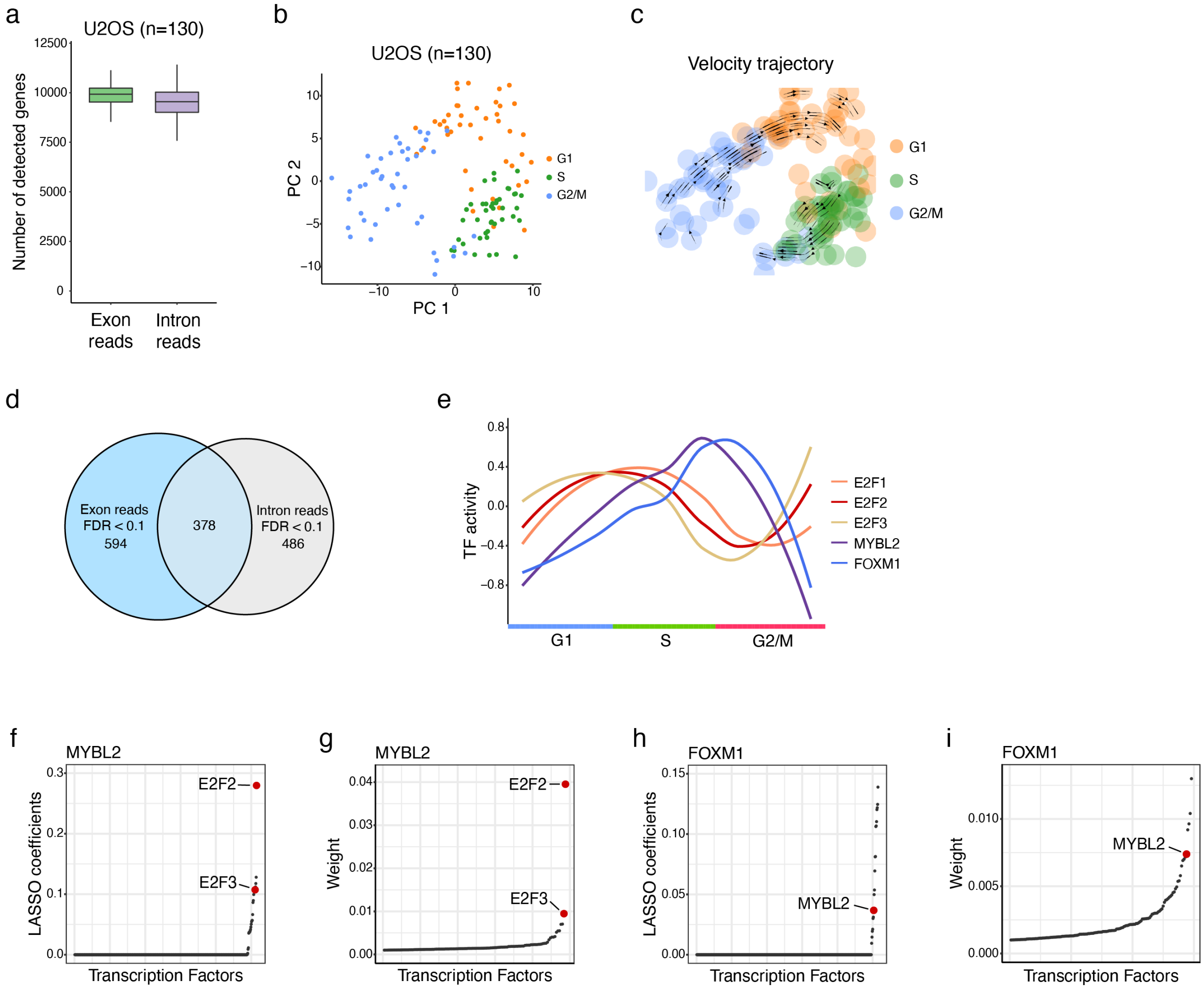

Supplementary figure 9. Cell cycle analysis on U2OS cell line. (**a**) The number of detected genes by exon reads or intron reads in single U2OS cells by snapTotal-seq. Center lines show median, box limits show the upper and lower quartiles, and whiskers show the 1.5× interquartile range (IQR). (**b**) PCA plot of U2OS cells. (**c**) The velocity trajectory projected by RNA velocity analysis. (**d**) Differential gene expression analyses identifying the genes with significant changes at the spliced RNA or unspliced RNA level along cell cycle in U2OS cell line. (**e**) The activities of different TF modules along cell cycle. (**f-g**) Identifying the regulatory relationships between G1 TFs and *MYBL2* using LASSO regression (**f**) or GENIE3 (**g**). The direct regulatory links (colored in red) were identified by ChIP-seq verification. (**h-i**) Identifying the regulatory relationship between *MYBL2* and *FOXM1* using LASSO regression (**h**) or GENIE3 (**i**). The direct regulatory links (colored in red) were identified by ChIP-seq verification.

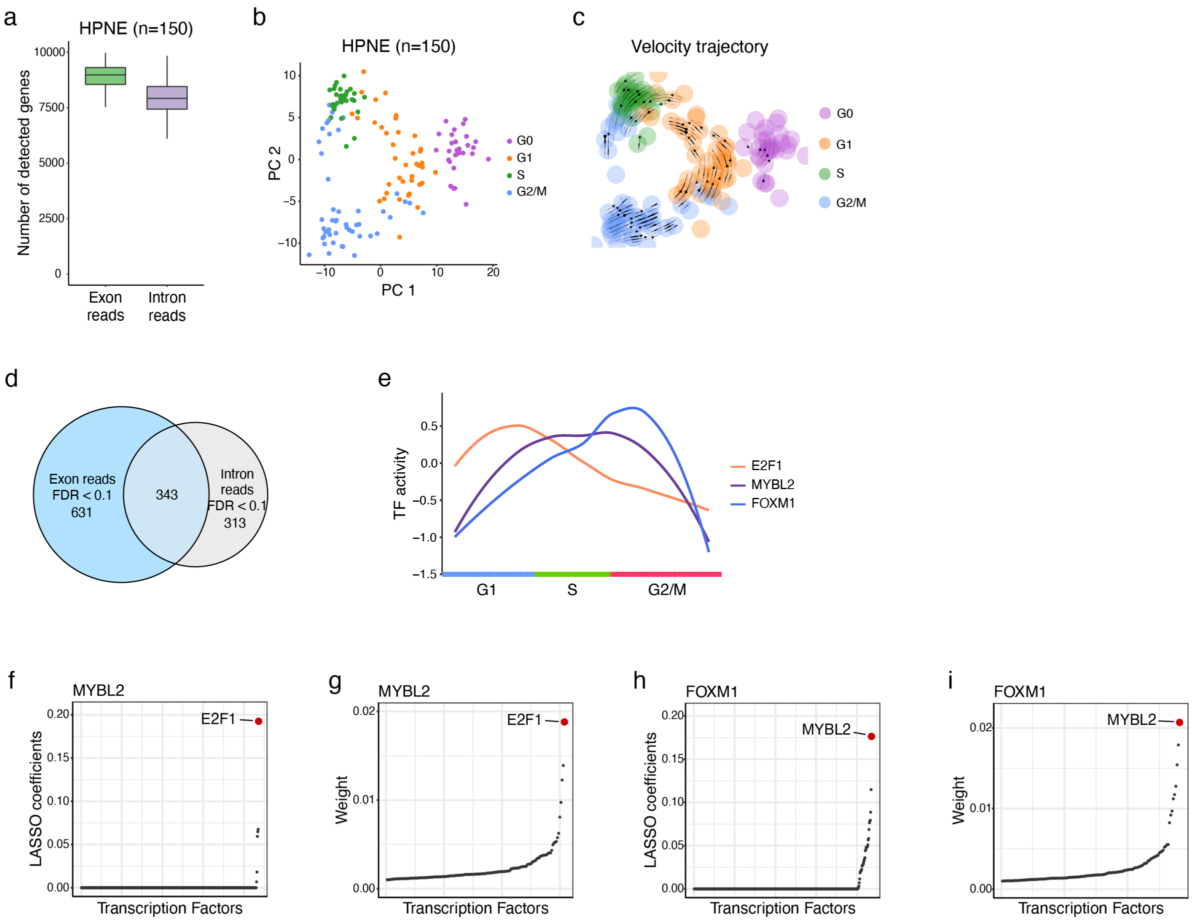

Supplementary figure 10. Cell cycle analysis on HPNE cell line. (**a**) The number of detected genes by exon reads or intron reads in single HPNE cells by snapTotal-seq. Center lines show median, box limits show the upper and lower quartiles, and whiskers show the 1.5× interquartile range (IQR). (**b**) PCA plot of HPNE cells. (**c**) The velocity trajectory projected by RNA velocity analysis. (**d**) Differential gene expression analyses identifying the genes with significant changes at the spliced RNA or unspliced RNA level along cell cycle in HPNE cell line. (**e**) The activities of different TF modules along cell cycle. (**f-g**) Identifying the regulatory relationships between *E2F1* and *MYBL2* using LASSO regression (**f**) or GENIE3 (**g**). The direct regulatory links (colored in red) were identified by ChIP-seq verification. (**h-i**) Identifying the regulatory relationship between *MYBL2* and *FOXM1* using LASSO regression (**h**) or GENIE3 (**i**). The direct regulatory links (colored in red) were identified by ChIP-seq verification. All cells (including G0 cells) were used in LASSO regression and GENIE3 analysis to increase the power of the covariation analyses.

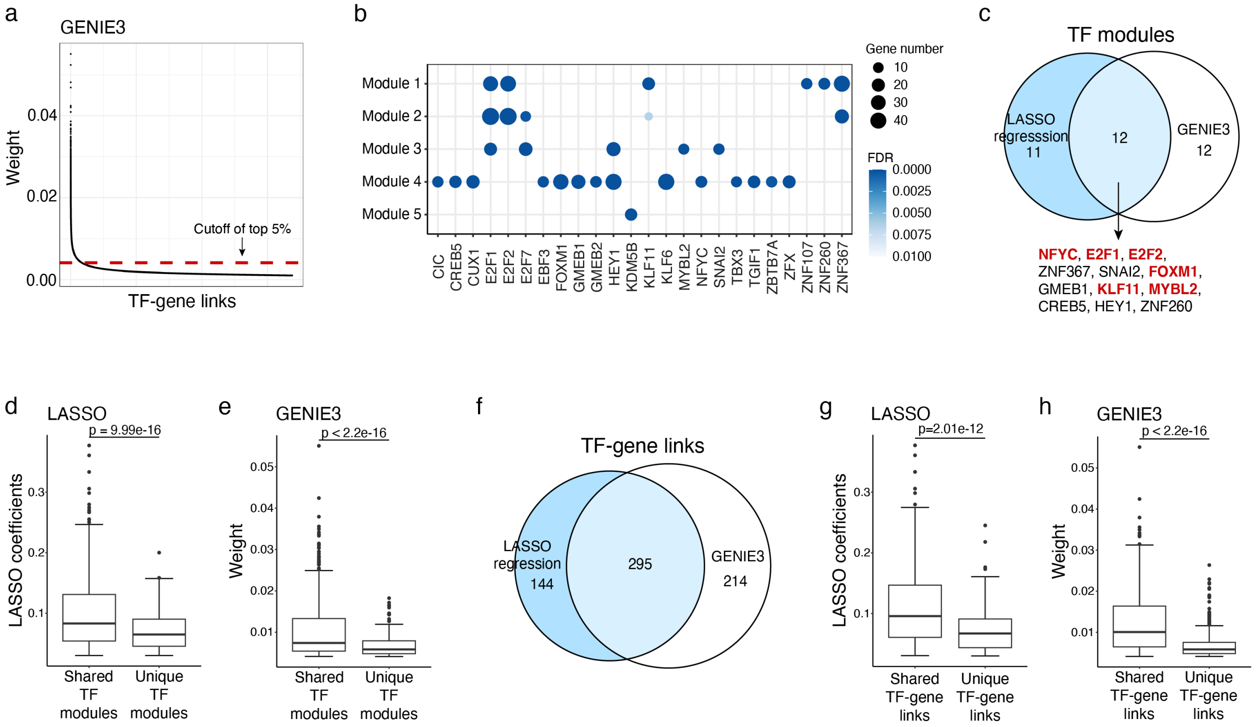

Supplementary figure 11. Infer TF modules in cell cycle regulation using GENIE3. (**a**) The ranking of TF-gene links based on their weight. The top 5% TF-gene links were considered as the ones with significance. (**b**) TFs whose associated genes are significantly enriched in Type I kinetic modules. Statistical significance was determined using one-sided Fisher's exact test and FDR was calculated using Benjamini-Hochberg procedure. (**c**) The comparison between the TF modules identified by LASSO regression and GENIE3 algorithm. The TF modules verified by published ChIP-seq data and/or motif enrichment analysis were colored in red. (**d**) The boxplots of the LASSO coefficients of the TF modules identified by both LASSO regression and GENIE3 algorithm, as well as those only identified by LASSO regression. (**e**) The boxplots of the weight of the TF modules identified by both LASSO regression and GENIE3 algorithm, as well as those only identified using GENIE3. (**f**) The comparison between the TF-gene links of the shared TF modules identified by LASSO regression and GENIE3 algorithm. (**g**) The boxplots of the LASSO coefficients of the TF-gene links identified by both LASSO regression and GENIE3 algorithm, as well as those only identified by LASSO regression. (**h**) The boxplots of the weight of the TF-gene links identified by both LASSO regression and GENIE3 algorithm, as well as those only identified using GENIE3. For all boxplots, center lines show median, box limits show the upper and lower

quartiles, and whiskers show the 1.5× interquartile range (IQR). *P* values were calculated using two-sided Student's t-test.

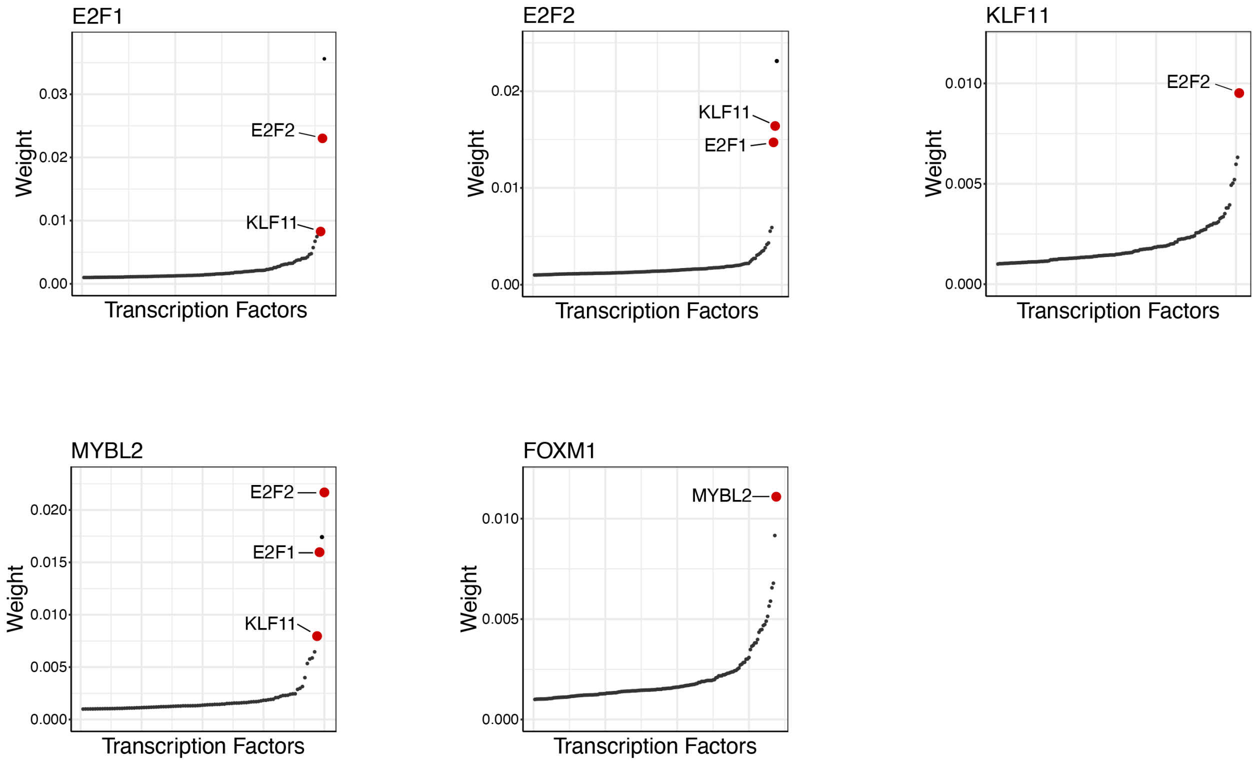

Supplementary figure 12. Infer TF-TF regulatory links in cell cycle regulation using GENIE3. The regulatory links between different TFs were established by correlating the changes in the unspliced RNA of the TF of interest with the changes in the spliced RNA of the other TFs using GENIE3. The regulatory links identified by LASSO regression and verified by ChIP-seq were colored in red. The results of LASSO regression and the ChIP-seq peaks were plotted in **Figure 3**.

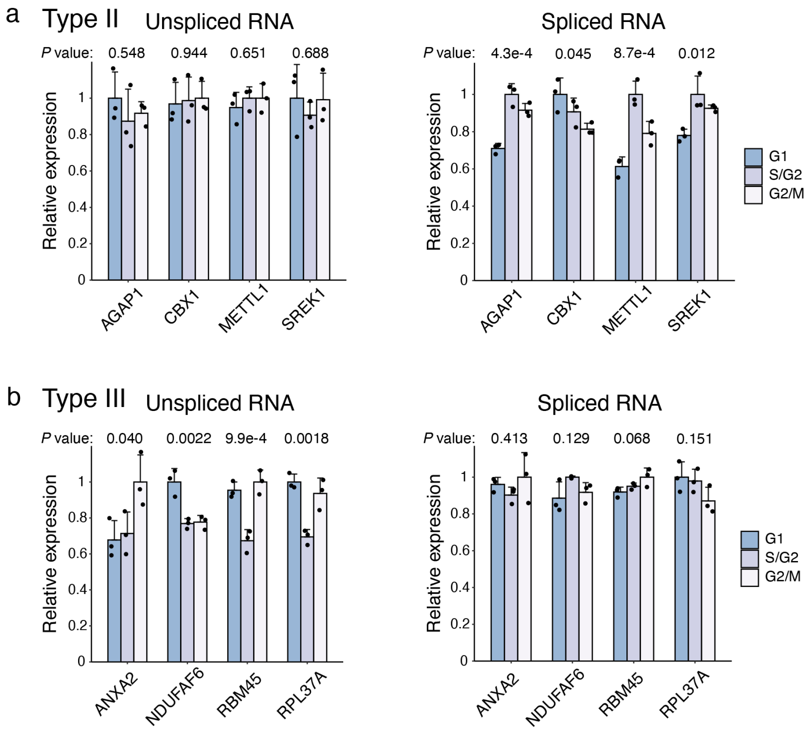

Supplementary figure 13. qRT-PCR validation Type II CCGs (**a**) and Type III CCGs (**b**). Three biological replicates were performed for each measurement. The mean and standard deviation were shown. One-way ANOVA was performed and *P* values were shown in the figure. Source data are provided as a Source Data file.

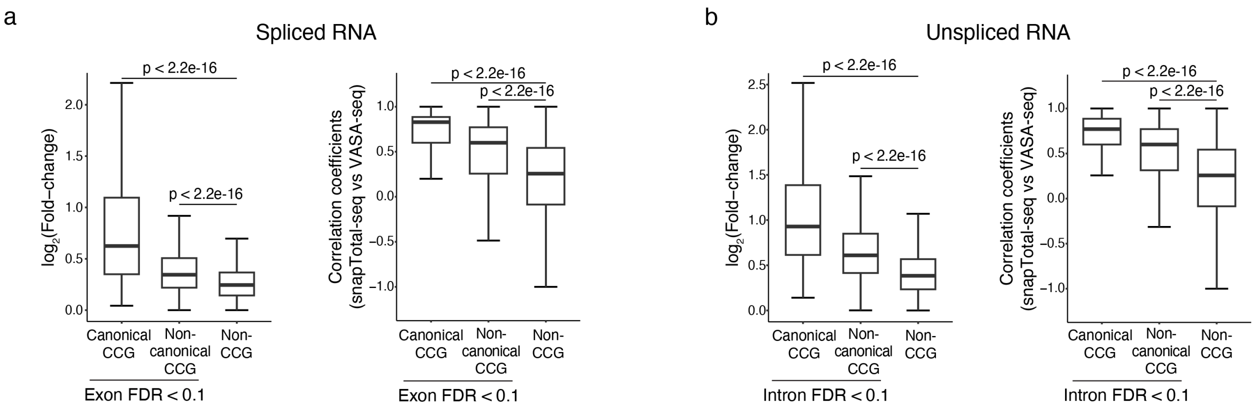

Supplementary figure 14. Cell-cycle-dependent expression of noncanonical CCGs in VASA-seq data. (**a**) Cell-cycle-dependent expression of CCGs at the spliced RNA level in VASA-seq data. Left panel, the boxplot of $\log_2$(fold-change) in gene expression across cell cycle of canonical CCGs and noncanonical CCGs in VASA-seq data. Right panel, the correlation coefficients between the cell-cycle dependent expression patterns identified in our data and VASA-seq data. (**b**) Cell-cycle-dependent expression of CCGs at the unspliced RNA level in VASA-seq data. Left panel, the boxplot of $\log_2$(fold-change) in gene expression across cell cycle of canonical CCGs and noncanonical CCGs in VASA-seq data. Right panel, the correlation coefficients between the cell-cycle dependent expression patterns identified in our data and VASA-seq data. Additional information is provided in **Supplementary note 1**. For all boxplots, center lines show median, box limits show the upper and lower quartiles, and whiskers show the 1.5× interquartile range (IQR). *P* values were calculated using two-sided Student's t-test.

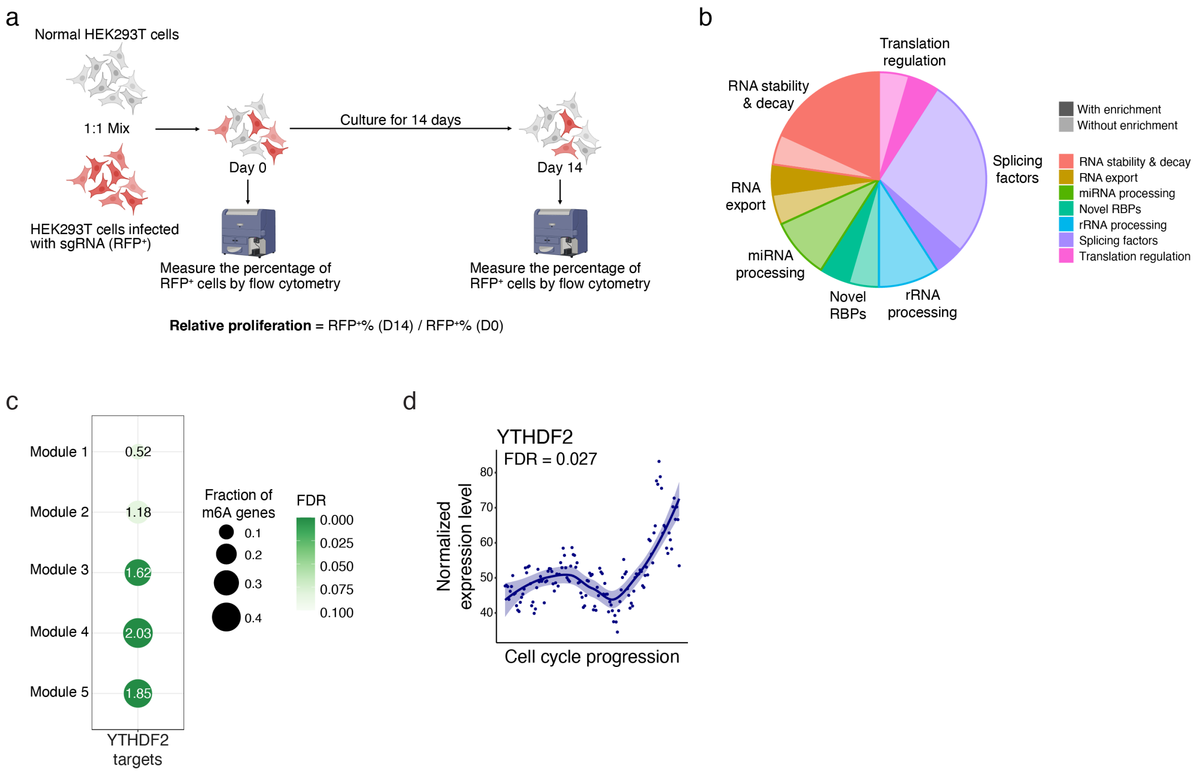

Supplementary figure 15. Investigate the roles of RBPs in cell proliferation. (**a**) The experimental scheme to examine the effects of the genes of interest on cell proliferation. Briefly, the transduced cells were approximately 1:1 mixed with uninfected cells. The initial percentage (referred to as day 0) of RFP^+ cells in the mixed cell population was measured by using flow cytometry. The mixed cell population was further cultured for 14 days, and the percentage of RFP^+ cells was measured by using flow cytometry after 14 days of culturing to determine the effects of the target gene on cell proliferation. The relative proliferation was calculated as RFP^+% (day 14) / RFP^+% (day 0). The plot was created with BioRender.com. (**b**) The functional categorization of RBPs with significant gene expression changes along cell cycle. (**c**) The significant enrichment of the target genes of YTHDF2 in the Type II CCGs with m6A modification. The statistical significance of enrichment is determined by Fisher's exact test, and FDR was calculated using Benjamini-Hochberg procedure. The enrichment fold in each module is labeled. (**d**) The mature RNA based gene expression dynamics of *YTHDF2* along the cell cycle. The smoothed curves were derived by using Loess function. The center line represents the average, and shade represents 0.95 confidence interval. Figure S15/panel a Created with BioRender.com released under a Creative Commons Attribution-NonCommercial-NoDerivs 4.0 International license.

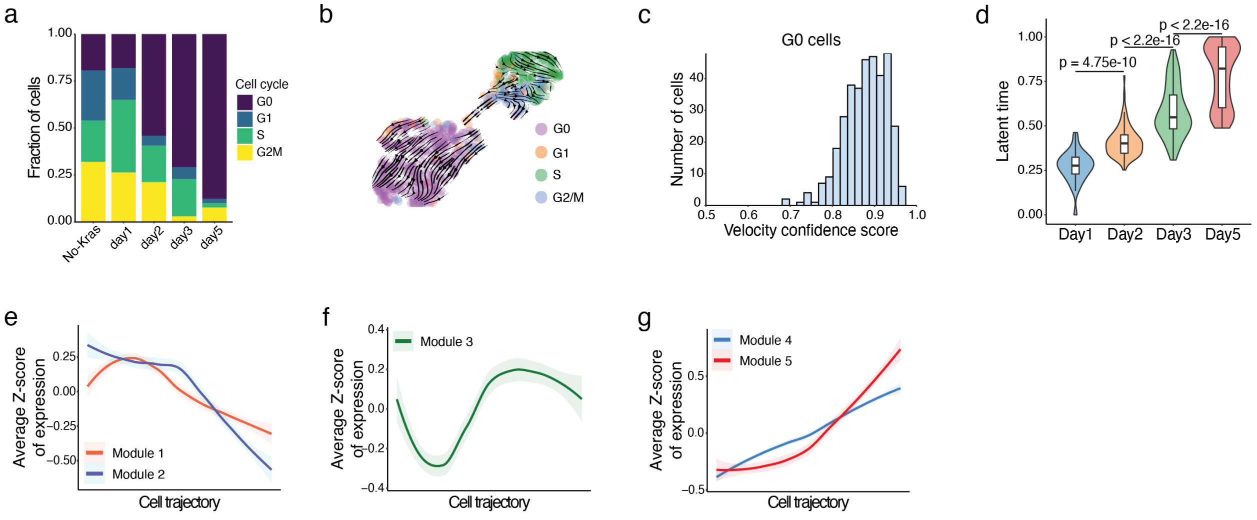

Supplementary figure 16. Gene expression dynamics during oncogene-induced senescence. (**a**) The fraction of cells at different cell cycle stages along the oncogene-induced senescence. Source data are provided as a Source Data file. (**b**) The projected velocity trajectory of oncogene-induced senescence by RNA velocity analysis. The cells are colored by the cell cycle stages. (**c**) The distribution of velocity confidence scores of all G0 cells. (**d**) The inferred latent time of the cells from different time points. *P* values calculated using two-sided Student's t-test. (**e-g**) The smoothed curves of the transcriptional dynamics along the latent time of each module. The smoothed curves were derived by using loess function. The center line represents the average, and shade represents 0.95 confidence interval.

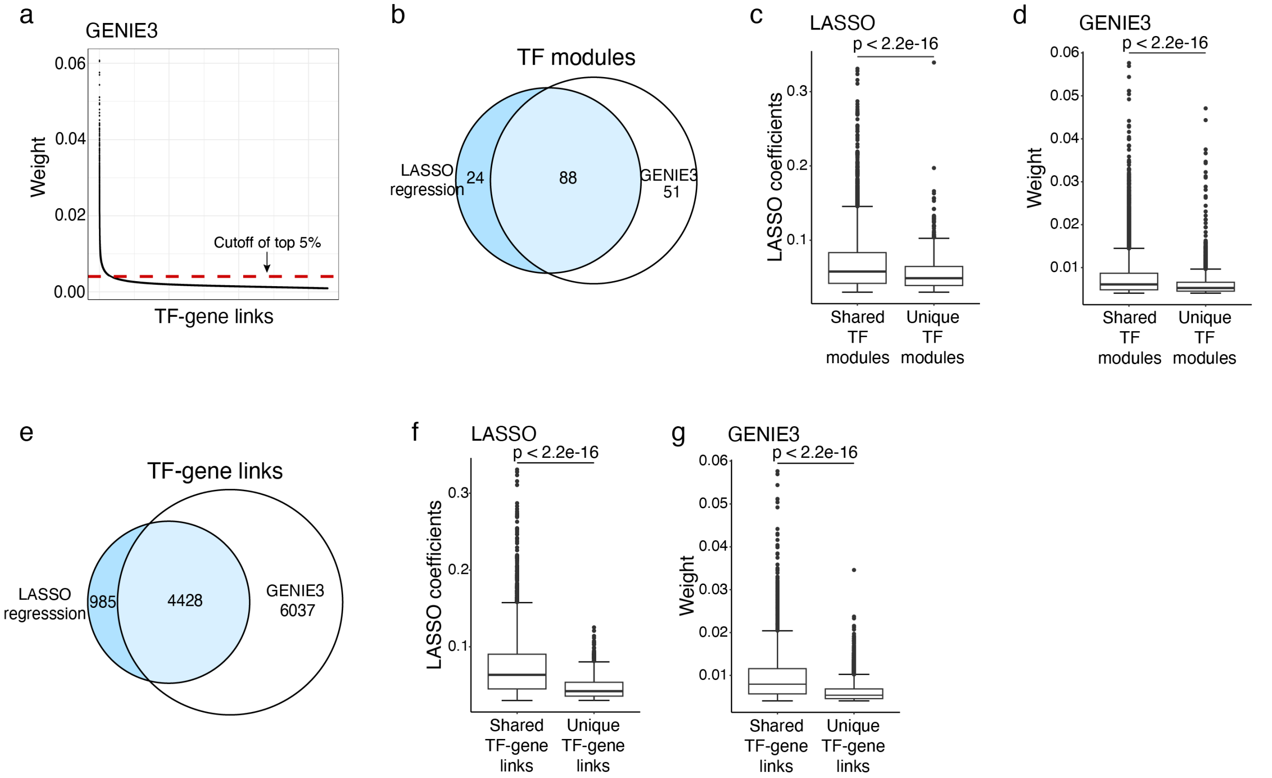

Supplementary figure 17. Infer TF modules in oncogene-induced senescence using GENIE3. (**a**) The ranking of TF-gene links based on their weight. The top 5% TF-gene links were considered as the ones with significance. (**b**) The comparison between the TF modules identified by LASSO regression and GENIE3 algorithm. (**c**) The boxplots of the LASSO coefficients of the TF modules identified by both LASSO regression and GENIE3 algorithm, as well as those only identified by LASSO regression. (**d**) The boxplots of the weight of the TF modules identified by both LASSO regression and GENIE3 algorithm, as well as those only identified using GENIE3. (**e**) The comparison between the TF-gene links of the shared TF modules identified by LASSO regression and GENIE3 algorithm. (**f**) The boxplots of the LASSO coefficients of the TF-gene links identified by both LASSO regression and GENIE3 algorithm, as well as those only identified by LASSO regression. (**g**) The boxplots of the weight of the TF-gene links identified by both LASSO regression and GENIE3 algorithm, as well as those only identified using GENIE3. For all boxplots, center lines show median, box limits show the upper and lower quartiles, and whiskers show the 1.5× interquartile range (IQR). *P* values were calculated using two-sided Student's t-test.

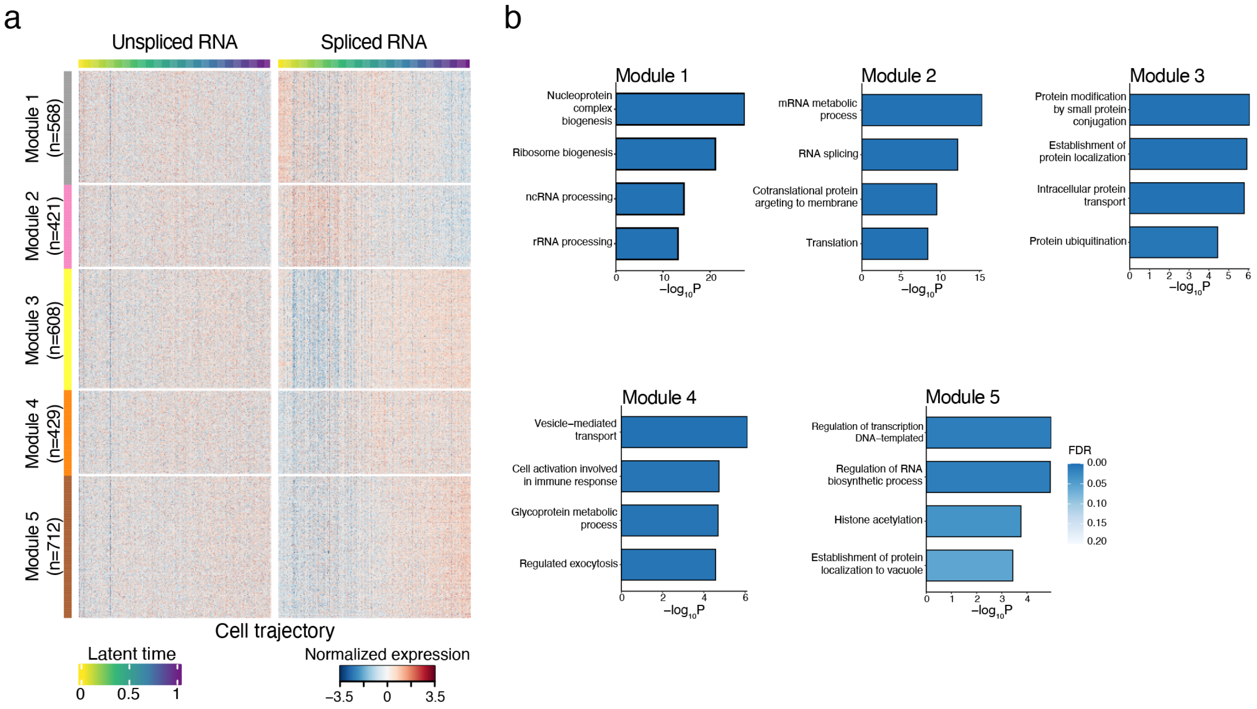

Supplementary figure 18. Functional analysis on Type II DEGs of oncogene-induced senescence. (**a**) The gene expression heatmap of the genes with significant changes at the mature RNA level only (Type II DEGs) along the latent time. (**b**) The GO functional enrichment for each kinetic module.

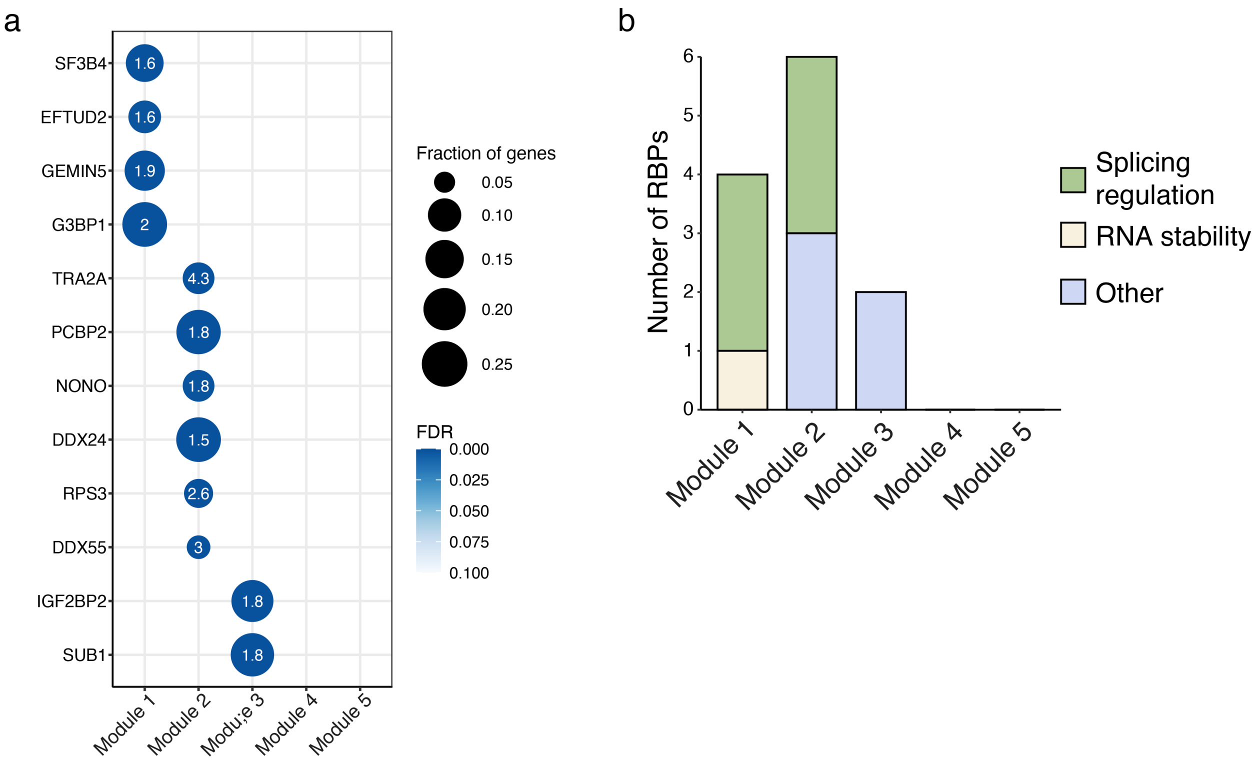

Supplementary figure 19. Enrichment analysis on the target genes of RBPs and Type II DEGs. (**a**) The significant enrichment of the target genes of RBPs with Type II DEGs. The RBPs need to meet the following criteria to be considered as significantly enriched: 1) The binding targets are significantly overrepresented (FDR < 0.1) in at least one of these modules; 2) The expression of the RBPs needs to be correlated (r > 0.3 or r < -0.3) with the corresponding kinetic module. (**b**) The functional categorization of RBPs with significant enrichment in Type II DEGs.

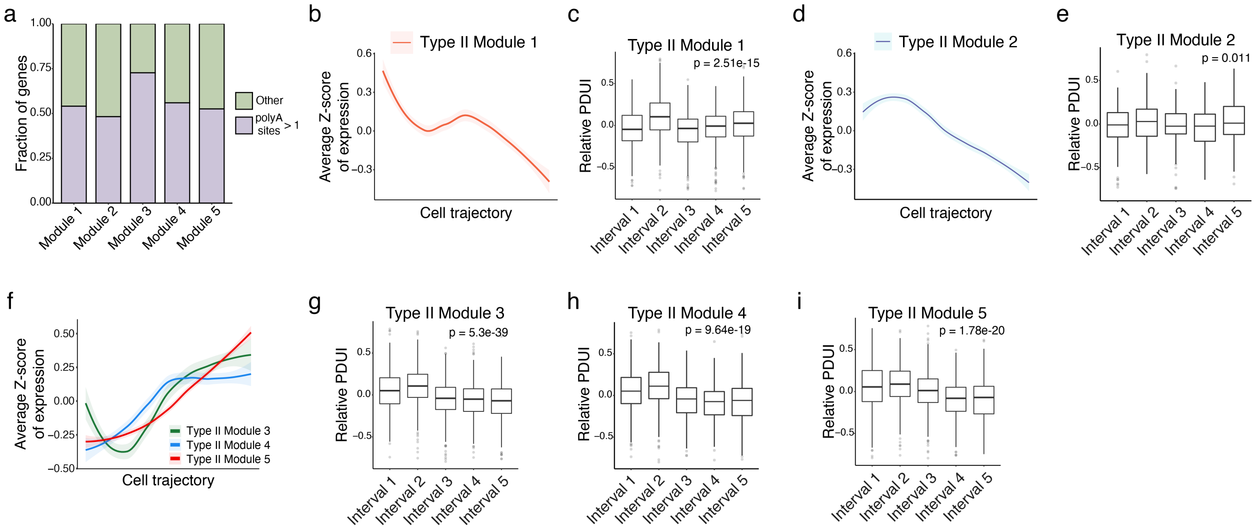

Supplementary figure 20. Alternative polyadenylation analysis on Type II DEGs. (**a**) The fraction of genes with multiple polyA sites in each Type II kinetic module. Source data are provided as a Source Data file. (**b**) The smoothed gene expression curves along the cell trajectory of oncogene-induced senescence for Type II Module 1. (**c**) The changes in polyA site usage of the genes in Type II Module 1 along the oncogene-induced senescence. (**d**) The smoothed gene expression curve along the latent time for Type II Module 2. (**e**) The changes in polyA site usage of the genes in Type II Module 2 along the oncogene-induced senescence. (**f**) The smoothed gene expression curves along the cell trajectory of oncogene-induced senescence for Type II Module 3, 4 and 5. (**g-i**) The changes in polyA site usage of the genes in Type II Module 3, 4 and 5 along the oncogene-induced senescence. PDUI: percentage of distal polyA site usage index. The relative PDUI was calculated by dividing the PDUI in each interval by the average PDUI across five intervals, followed by log-transformation. Therefore, the positive relative PDUI represents the increased usage of distal polyA sites, and the negative relative PDUI represents the decreased usage of distal polyA sites. For all boxplots, center lines show median, box limits show the upper and lower quartiles, and whiskers show the 1.5× interquartile range (IQR). One-way ANOVA was performed and *P* values were shown in the figure. The smoothed curves were derived by using loess function. The center line represents the average, and shade represents 0.95 confidence interval.

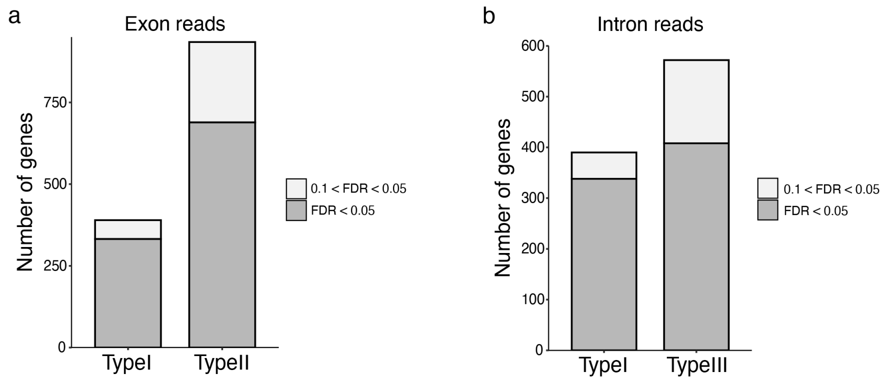

Supplementary figure 21. CCGs with different FDR cutoff. (**a**) The number of Type I and Type II CCGs with different FDR cutoffs in exon-based analysis. (**b**) The number of Type I and Type III CCGs with different FDR cutoffs in intron-based analysis. Additional information is provided in **Supplementary note 3**.

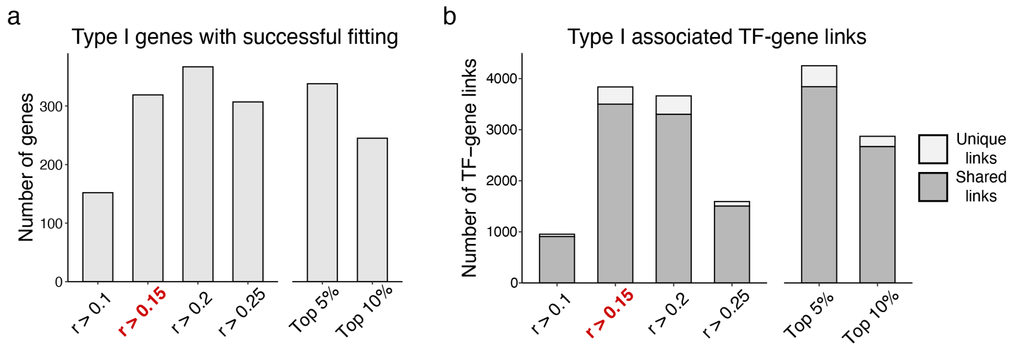

Supplementary figure 22. Evaluation of the effects of different parameters on LASSO regression model. (**a**) The number Type I genes with successful fitting under different parameters. (**b**) The number of TF-gene links identified under different parameters. The parameter used in our analysis is highlighted in red. Additional information is provided in **Supplementary note 4**.

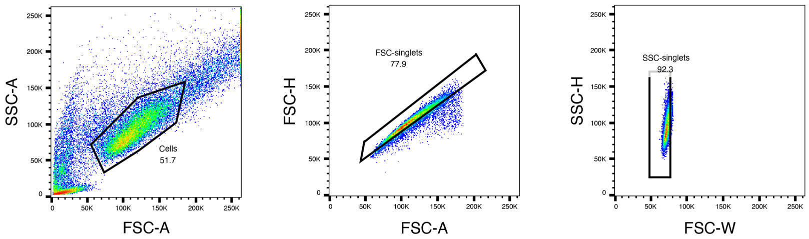

Supplementary figure 23. Illustration of gating strategy. The first gating is size selection to remove the debris. The second and third gatings are singlet selection to remove potential doublets.

References

1 Richart, L. *et al.* BPTF is required for c-MYC transcriptional activity and in vivo tumorigenesis. *Nat Commun* **7**, 10153, doi:10.1038/ncomms10153 (2016).

2 Gao, Q. *et al.* Knockdown of RREB1 inhibits cell proliferation via enhanced p16 expression in gastric cancer. *Cell Cycle* **20**, 2465-2475, doi:10.1080/15384101.2021.1987676 (2021).

3 Chen, D., Hinkley, C. S., Henry, R. W. & Huang, S. TBP dynamics in living human cells: constitutive association of TBP with mitotic chromosomes. *Mol Biol Cell* **13**, 276-284, doi:10.1091/mbc.01-10-0523 (2002).

4 Iwasaki, O. *et al.* Interaction between TBP and Condensin Drives the Organization and Faithful Segregation of Mitotic Chromosomes. *Mol Cell* **59**, 755-767, doi:10.1016/j.molcel.2015.07.007 (2015).

5 Teves, S. S. *et al.* A stable mode of bookmarking by TBP recruits RNA polymerase II to mitotic chromosomes. *Elife* **7**, doi:10.7554/eLife.35621 (2018).
