# Supplementary material for: Single-cell total-RNA profiling unveils regulatory hubs of transcription factors
Source: Nat Commun. 2024 Jul 15;15:5941. doi: 10.1038/s41467-024-50291-3 (PMC11251146; doi:10.1038/s41467-024-50291-3)
Supplement: Supplementary file 3 — Description of Additional Supplementary Files [file 41467_2024_50291_MOESM3_ESM.pdf]

Description of Additional Supplementary Files

File name: Supplementary Data 1
Description: Differentially expressed genes along the cell cycle in HEK293T cell line.

File name: Supplementary Data 2
Description: log2FC in gene expression along cell cycle.

File name: Supplementary Data 3
Description: Module assignment for Type I and II CCGs.

File name: Supplementary Data 4
Description: Verified cell cycle related TF modules in HEK293T cell line.

File name: Supplementary Data 5
Description: Candidate TF-TF regulatory relationships identified by LASSO regression.

File name: Supplementary Data 6
Description: Differentially expressed genes during oncogene-induced senescence (OIS).

File name: Supplementary Data 7
Description: Module assignment for Type I and II DEGs.

File name: Supplementary Data 8
Description: TF hubs identified in OIS.

File name: Supplementary Data 9
Description: Verified TF modules in OIS.

File name: Supplementary Data 10
Description: Sequence of the primers used for qRT-PCR.

File name: Supplementary Data 11
Description: sgRNA sequence used in knock down experiments.

File name: Supplementary Data 12
Description: Sequence of DNA oligos used in snapTotal-seq.
