# Supplementary material for: Single-cell total-RNA profiling unveils regulatory hubs of transcription factors
Source: Nat Commun. 2024 Jul 15;15:5941. doi: 10.1038/s41467-024-50291-3 (PMC11251146; doi:10.1038/s41467-024-50291-3)
Supplement: Supplementary file 16 — Reporting Summary [file 41467_2024_50291_MOESM16_ESM.pdf]

nature portfolio

Corresponding author(s): Chenghang Zong

Last updated by author(s): Mar 11, 2024

Reporting Summary

Nature Portfolio wishes to improve the reproducibility of the work that we publish. This form provides structure for consistency and transparency in reporting. For further information on Nature Portfolio policies, see our Editorial Policies and the Editorial Policy Checklist.

Statistics

For all statistical analyses, confirm that the following items are present in the figure legend, table legend, main text, or Methods section.

n/a	Confirmed	
☐	☒	The exact sample size (*n*) for each experimental group/condition, given as a discrete number and unit of measurement
☐	☒	A statement on whether measurements were taken from distinct samples or whether the same sample was measured repeatedly
☐	☒	The statistical test(s) used AND whether they are one- or two-sided<br>*Only common tests should be described solely by name; describe more complex techniques in the Methods section.*
☒	☐	A description of all covariates tested
☐	☒	A description of any assumptions or corrections, such as tests of normality and adjustment for multiple comparisons
☐	☒	A full description of the statistical parameters including central tendency (e.g. means) or other basic estimates (e.g. regression coefficient) AND variation (e.g. standard deviation) or associated estimates of uncertainty (e.g. confidence intervals)
☐	☒	For null hypothesis testing, the test statistic (e.g. *F*, *t*, *r*) with confidence intervals, effect sizes, degrees of freedom and *P* value noted<br>*Give P values as exact values whenever suitable.*
☒	☐	For Bayesian analysis, information on the choice of priors and Markov chain Monte Carlo settings
☒	☐	For hierarchical and complex designs, identification of the appropriate level for tests and full reporting of outcomes
☐	☒	Estimates of effect sizes (e.g. Cohen's *d*, Pearson's *r*), indicating how they were calculated

Our web collection on statistics for biologists contains articles on many of the points above.

Software and code

Policy information about availability of computer code

Data collection	Fluorescence activated cell sorting (FACS) was performed on BD Aria II.<br>The libraries were sequenced on NextSeq 500 machine with customized sequencing primers as follows: Read 1 sequencing primer: ACA CTC TTT CCC TAC ACG ACG CTC TTC CGA TCT, Read 2 sequencing primer: AGA GGT GAG TGA GTG ATG GTT GAG GAT GTG TGG AG, Index i5 sequencing primer: AGA TCG GAA GAG CGT CGT GTA GGG AAA GAG TGT, Index i7 sequencing primer: CTC CAC ACA TCC TCA ACC ATC ACT CAC TCA CCT CT.
Data analysis	Read 1 was mapped to human genome assembly (GRCh37) by using STAR (v2.5.3a). The uniquely mapped reads were then mapped to the gene annotations of GENCODE (v19) by using htseq-count (v0.13.5) with 'intersection-strict' mode and with option '--stranded=no'. To discern the amplicons from exons and introns, the 'transcript' feature and the 'exon' feature were used respectively. The UMI sequences (the first five bases of read 2) of the reads mapped to the gene regions (either the 'transcript' feature or the 'exon' feature) were extracted. The reads were then grouped by the UMI sequence and the gene that they were mapped to. If all the reads within the group were mapped to the exon regions of the corresponding gene, the original amplicon was classified as an exonic amplicon. Otherwise, the original amplicon was classified as an intronic amplicon. For the analysis on Smart-seq-total data, cutadapt (v3.4) was used to remove poly(A) sequences.<br>Normalization and PCA was performed using Seurat (v4.0.3). Gene expression matrices (exon and intron) were converted to HDF5 format using SeuratDisk (v0.0.0.9019) for RNA velocity analysis. RNA velocity analysis was carried out using scvelo (v0.2.3). Cell cycle analysis was performed using reCAT package. Differential gene expression analysis was performed using tradeSeq (v1.6.0). Gene ontology enrichment analysis was performed using goseq (v1.44.0). The known cell cycle gene list was obtained from the cell cycle pathway in Gene Ontology database, Reactome database and Cyclebase. LASSO regression was performed using glmnet (v4.1.7). The visualization of the TF regulatory network was performed using Cytoscape (v3.8.2).<br>For ChIP-seq analysis, the peak files were downloaded from CistromeDB. The samples with median mapping quality ≥ 25, unique mapping rate ≥ 0.6, PCR bottleneck coefficient ≥ 0.8, fraction of reads in peaks ≥ 0.01, peak number (fold > 10) ≥ 150, and high consistence with DNase-seq

data (percentage of top 5k peaks overlapped with DNase-seq ≥ 0.85) were kept. The rest of datasets were not used due to potentially low quality. The qualified datasets were then grouped based on their target TFs. For each TF, the peaks (fold > 10) identified in corresponding datasets were merged, and the peak annotation was performed using HOMER. The binding targets were identified if the peak was within 1kb or 5kb from transcription start site. These two different cutoffs corresponded to the scenarios of binding to the promoter region or binding to the enhancer region, respectively.
For ChIP-seq peak visualization, the raw data or the processed data were downloaded. The processed data were directly used for visualization in Integrative Genomics Viewer (IGV). Raw data were mapped to human genome assembly (GRCh37) using Bowtie2 (v2.2.5) after removing the adapter sequences with Cutadapt (v3.4). After deduplication using picard (v2.26.11), BAM files were converted to bigwig files using bamCoverage function in deepTools package (v3.5.1). Bigwig files were then used for visualization in IGV. Motif enrichment analysis was carried out within 500bp upstream of TSS or 5kb around the TSS using RcisTarget package(v1.12.0).
The code used for analyzing snapTotal-seq data is available at https://github.com/zonglab/snapTotal-seq.git

For manuscripts utilizing custom algorithms or software that are central to the research but not yet described in published literature, software must be made available to editors and reviewers. We strongly encourage code deposition in a community repository (e.g. GitHub). See the Nature Portfolio guidelines for submitting code & software for further information.

Data

Policy information about availability of data

All manuscripts must include a data availability statement. This statement should provide the following information, where applicable:

- Accession codes, unique identifiers, or web links for publicly available datasets
- A description of any restrictions on data availability
- For clinical datasets or third party data, please ensure that the statement adheres to our policy

The raw data of Smart-seq3 were obtained from ArrayExpress: E-MTAB-8735 at EMBL-EBI. The raw data of CEL-Seq2 were obtained from the Gene Expression Omnibus: GSE132044 (SRR9167461, SRR9167462, SRR9171229, SRR9171230). The raw data of VASA-plate were obtained from the Gene Expression Omnibus: GSE176588. The raw data of Smart-seq-total were obtained from the Gene Expression Omnibus: GSE151334. The raw data and the processed data sets generated in this study are available at the Gene Expression Omnibus under the accession number: GSE202126.
For ChIP-seq visualization, the following datasets were used: E2F1, GSM2132552; E2F2, ENCFF826PYA; KLF11, GSE59703; MYBL2, ENCFF487KQK; REL, GSE55105; NFKB2, GSE55105; KLF10, ENCFF437PEU; STAT2, ENCFF133YDB; ELF1, ENCFF937HON; GTF2B, GSE71848; STAT1, GSE43036; ATF4, GSE69309; ATF3, ENCFF783IOJ; CEBPG, ENCFF182SSK.

Research involving human participants, their data, or biological material

Policy information about studies with human participants or human data. See also policy information about sex, gender (identity/presentation), and sexual orientation and race, ethnicity and racism.

Reporting on sex and gender	N/A
Reporting on race, ethnicity, or other socially relevant groupings	N/A
Population characteristics	N/A
Recruitment	N/A
Ethics oversight	N/A

Note that full information on the approval of the study protocol must also be provided in the manuscript.

Field-specific reporting

Please select the one below that is the best fit for your research. If you are not sure, read the appropriate sections before making your selection.

☒ Life sciences ☐ Behavioural & social sciences ☐ Ecological, evolutionary & environmental sciences

For a reference copy of the document with all sections, see nature.com/documents/nr-reporting-summary-flat.pdf

Life sciences study design

All studies must disclose on these points even when the disclosure is negative.

Sample size	No statistical methods were used to pre-determine the sample size. All in vitro experiments were repeated at least three times based on common practices.
Data exclusions	No data were excluded.
Replication	All replication attempts were successful.

Randomization	The cell lines were randomly seeded in all related experiments.
Blinding	No experiments need blinding for analysis.

Reporting for specific materials, systems and methods

We require information from authors about some types of materials, experimental systems and methods used in many studies. Here, indicate whether each material, system or method listed is relevant to your study. If you are not sure if a list item applies to your research, read the appropriate section before selecting a response.

Materials & experimental systems

n/a	Involved in the study
☒	☐ Antibodies
☐	☒ Eukaryotic cell lines
☒	☐ Palaeontology and archaeology
☒	☐ Animals and other organisms
☒	☐ Clinical data
☒	☐ Dual use research of concern
☒	☐ Plants

Methods

n/a	Involved in the study
☒	☐ ChIP-seq
☐	☒ Flow cytometry
☒	☐ MRI-based neuroimaging

Eukaryotic cell lines

Policy information about cell lines and Sex and Gender in Research

Cell line source(s)	HEK293T(CRL-3216), hTERT-HPNE(CRL-4023) and U2-OS(HTB-96) cell lines were obtained from ATCC.
Authentication	Authentication was performed at BCM cell line cores based on morphology and karyotyping.
Mycoplasma contamination	Tested negative for mycoplasma contamination.
Commonly misidentified lines (See ICLAC register)	N/A

Plants

Seed stocks	N/A
Novel plant genotypes	N/A
Authentication	N/A

Flow Cytometry

Plots

Confirm that:

☒ The axis labels state the marker and fluorochrome used (e.g. CD4-FITC).

☒ The axis scales are clearly visible. Include numbers along axes only for bottom left plot of group (a 'group' is an analysis of identical markers).

☒ All plots are contour plots with outliers or pseudocolor plots.

☒ A numerical value for number of cells or percentage (with statistics) is provided.

Methodology

Sample preparation	Cells were trypsinized and resuspended in PBS.

Instrument	BD FACSAriaII P6950002
Software	BD FACSDiva version 8.0.1
Cell population abundance	Post-sort was performed, and the purity of target population was > 99.9%.
Gating strategy	The first gating is size selection to remove the debris; The second and third gatings were singlet selection to remove potential doublets.

☒ Tick this box to confirm that a figure exemplifying the gating strategy is provided in the Supplementary Information.
